# Young people with migration experience and their (non) encounters with Swedish sexual and reproductive health services and information: An explorative study

**DOI:** 10.1016/j.jmh.2024.100270

**Published:** 2024-10-05

**Authors:** Nada Amroussia, Malin Lindroth, Catrine Andersson

**Affiliations:** a Centre for Sexology and Sexuality Studies, Department of Social Work, Faculty of Health and Society, Malmö University, Nordenskiöldsgatan 1, Malmö 211 19, Sweden; b Department of Behavioural Science, Faculty of Health Sciences, Oslo Metropolitan University, Oslo, Norway

**Keywords:** Sexual and reproductive health and rights, Access to healthcare services, Healthcare service utilization, Migration, Young migrants, Young people with migration experience, Reflexive thematic analysis

## Abstract

Although a growing body of literature has focused on the experience of young people with migration experience with Swedish sexual and reproductive health (SRH) services, there is a lack of deep qualitative exploration. The study aims to explore the encounters of young people with migration experience with SRH services and their understandings of factors that affect their use of these services. The findings of this study were drawn from 18 interviews conducted between October 2021 and May 2023 in Southern Sweden. A combination of convenient and snowball sampling strategies was used. Participants included in the study self-identified as Middle Eastern, migrated to Sweden, and were aged between 17 and 26. Data were analyzed using reflexive thematic analysis approach.

Three themes were generated during the analysis. The first *SRH services: dual perceptions and experiences* shows how participants had ambivalent perceptions of SRH services, mainly the youth clinic. Some perceived the youth clinic as a stigmatized place associated with shame and SRH concerns like unwanted pregnancy and sexually transmitted infections, while others viewed the youth clinic as a safe space. The negative perceptions along with the difficulties with accessing the youth clinic contributed to low service use. The second *Sexuality education: an eye-opener or a joke?* reflects participants’ both positive and negative experiences and attitudes when receiving sexuality education in schools. The third *SRH information: beyond formal services and education* captures participants’ ways of accessing SRH information that go beyond information provided at the traditional SRH services and sexuality education in schools. These sources include the family, friends, and the internet. The study points to the need for multicomponent strategies to improve the accessibility of SRH services and draws attention to the importance of challenging norms related to Swedishness in sexuality education to foster the engagement of youth with migration experience and ensure their sexual citizenship.

## 1 Introduction

Young people with migration experience represent a particularly disadvantaged group in relation to sexual and reproductive health and rights (SRHR). For instance, previous research in high-income countries has pointed to the vulnerability of this group to violence, and their poor sexual and reproductive health (SRH) outcomes (Mason-Jones and Nicholson, 2018). Research has also pointed to the low levels of sexual health literacy among young people with migration experience, including limited knowledge of sexually transmitted infections (STIs) (Ngum Chi Watts et al., 2015; McMichael and Gifford, 2009; McMichael and Gifford, 2010) and limited knowledge of contraception (Ngum Chi Watts et al., 2014). Additionally, previous studies have highlighted various barriers to accessing SRHR related information as well as barriers to accessing and using SRH services among this group. These barriers include lack of knowledge about SRH services, distrust of the healthcare system (Salad et al., 2015), confidentiality concerns (McMichael and Gifford, 2009), perceiving the services as irrelevant and inappropriate (Afroz et al., 2021), shame and embarrassment (McMichael and Gifford, 2009; Salad et al., 2015), as well as taboos related to sexuality (Botfield et al., 2020). Several studies have also focused on their views on SRHR and SRH services, and highlighted the role of cultural beliefs and values in shaping these views (Afroz et al., 2021; Botfield et al., 2020). They have also pointed to the part that gender roles, and family members, primarily parents, play in shaping young migrants’ attitudes towards and use of, for example, contraception (Ngum Chi Watts et al., 2015; Ngum Chi Watts et al., 2014).

The vulnerabilities and barriers outlined above suggest that young people with migration experience might not fully enjoy their s*exual citizenship*. This concept expands the notion of citizenship beyond the legal, political, and social domains (Weeks, 1998) and implies claims to rights like the right to participate in sexual activity, the right to sexual and reproductive self-determination and the right to self-expression (Richardson, 2000). Sexual citizenship has common features with other claims to citizenship: enfranchisement, inclusion, belonging, equity, and justice (Weeks, 1998). It also defines the terms of exclusion (Richardson, 2004). Youth and young people's sexual citizenship might be on-hold due to “their lack of capital, authority, and resources” (12 p. 295) restricting their citizenship practices, including claiming their rights as sexual citizens (Talburt, 2018). This is particularly relevant for young people with migration experience whose rights of sexual citizens, as other migrant groups (Richardson, 2018), might be contested.

In Sweden, SRHR among young people with migration experience has received growing attention in the past few years. As in other studies in high-income countries, research conducted in Sweden has pointed to the vulnerability of this group. For instance, a study found that one-quarter of participants (young migrants, 16–29 years old) reported experiencing some form of sexual violence and that nearly four out of ten stated that they felt limited regarding with whom they can have an intimate relationship (Baroudi et al., 2021). Another recently published study, focusing on the self-reported sexual risk-taking behaviors among migrant youth (15–25 years old) in Sweden, showed the variability in engaging in sexual risk-taking practices depends on different factors including age, the country/region of origin, length of stay and religious affiliation. For instance, the prevalence of engaging in sex without a condom was higher among migrant youth who were older, coming from the Americas and Europe, and having lived longer in Sweden (Causevic et al., 2022).

Previous research in Sweden has also indicated that young people with migration experience face challenges in accessing and using SRH services. A study focusing on young migrants (16–29 years old) revealed that nearly one-sixth of participants did not use SRH services in the last year despite stating a need for care (Baroudi et al., 2021), whereas a related study showed that nearly one-third of participants (young migrants, 16–29 years old) reported needing SRH services during the last year, and only one-third of those participants reported fulfilling their needs (Baroudi et al., 2020). Lack of knowledge about the available services, long waiting times, language difficulties, financial difficulties, lack of trust towards the Swedish system, and previous negative encounters with healthcare systems were the most commonly mentioned reasons for refraining from using SRH services (Baroudi et al., 2020).

Although a growing body of literature has focused on this group's experiences with SRH services and information in Sweden, there is a paucity of research generating a deep qualitative exploration of these experiences. Such exploration is needed to improve the accessibility and quality of SRH services and information provided. A qualitative study conducted among Iraqi youth (18–24 years old) having migrated to Sweden drew attention to cultural barriers for accessing SRHR related information among this group (Flodström et al., 2011). Another qualitative study showed difficulties that unaccompanied minors’ (18–22 years old) SRHR, encountered in accessing SRHR-related information (Nordström and Agardh, 2021).

To investigate this gap in the literature and to deepen our understanding of SRHR issues among this group, the current study aims to explore the encounters of young people with migration experience with SRH services and their understandings of factors that affect their use of these services. We refer to the comprehensive definition of SRH services provided by the Guttmacher-Lancet commission report (Starrs et al., 2018), and adopted by the Swedish national SRHR policy (Folkhälsomyndigheten 2020). Accordingly, SRH services are defined as “accurate information and counselling on sexual and reproductive health, including evidence-based, comprehensive sexuality education; information, counselling, and care related to sexual function and satisfaction; prevention, detection, and management of sexual and gender-based violence and coercion; a choice of safe and effective contraceptive methods; safe and effective antenatal, childbirth, and postnatal care; safe and effective abortion services and care; prevention, management, and treatment of infertility; prevention, detection, and treatment of sexually transmitted infections, including HIV, and of reproductive tract infections; and prevention, detection, and treatment of reproductive cancers” (Starrs et al., 2018).

## 2 Material and methods

### 2.1 Study design

A qualitative methodology with an emergent design was used in the study (Guba and Lincoln, 1982). Individual interviews were employed as the study addresses a topic that might be considered sensitive by participants (Gill et al., 2008).

### 2.2 Study setting

The data collection was conducted in a city in Southern Sweden in Scania County. The total population of this region is around 1,4 million inhabitants. The percentage of foreign-born residents is slightly higher than the national percentage of 20 % in 2022 (Sweden, 2023) and amounted to 23 % in 2021 (RegionSkåne 2023). People from the Middle East represent one of the largest migrant groups in the region (RegionSkåne 2023), and in the biggest city in the region, Iraq and Syria are the most common countries of birth for foreign-born residents (Stad 2023).

#### 2.2.1 SRH services and SRH information in Sweden

Youth clinics represent the specific provider of SRH services to youth aged between 12 and 25 in Sweden (FSUM 2018). The services are free of charge for youth under the age of 18 and most of the clinics provide services free of charge for youth under the age of 25 (Åkerman, 2019). In addition to youth clinics, SRH services are also provided by other healthcare facilities such as primary healthcare centers and the school healthcare team. Examples of SRH services provided at primary healthcare centers for free include HIV/STI testing and contraceptive counselling (Åkerman, 2019), and the school healthcare teams offer immunization for human papilloma virus (Sweden 2023). Undocumented migrants are entitled to some SRH services, such as maternal healthcare, contraceptive counseling, and abortion care (Socialstyrelsen 2019).

With regards to SRH information, a national survey conducted in 2017 revealed that sexuality education in schools represented the third most common source of SRH information for youth in Sweden, after internet and friends (Sweden, 2017). In Sweden, sexuality education has been mandatory in schools since 1955 (Bredström et al., 2018). Since then, several reforms have affected its provision. For instance, in the 1970s, there was a shift from the abstinence approach towards the notion of sexual responsibility with providing education on contraceptive methods (Bolander, 2015). In 2011, there was a shift towards considering sexuality education, called sex and relationship education, an interdisciplinary subject that was supposed to be incorporated into all subjects and courses’ syllabi (Education), S.S.N.A.f 2011). Since 2022, sexuality education in Swedish schools has been called sexuality, consent, and relationships. The purpose is to put a stronger emphasis on the consent component (Skolverket. Sexualitet, samtycke och relationer, 2023).

### 2.3 Recruitment and data collection

The data collection was conducted between October 2021 and May 2023. Convenient and snowball sampling (Rapley, 2014) were employed. The recruitment procedure involved using social media and reaching out to non-governmental organizations working with migrants, schools, and youth centers. Participants included in this study self-identified as Middle Eastern, migrated to Sweden, and were aged 16 or above. A total of 18 individual semi-structured interviews were conducted by the first author. An interview guide was constructed, and it covered the following topics: 1) experiences with youth clinics and other SRH services including SRH information and sexuality education, and 2) views on sexuality and sexual experiences (Appendix A). The current paper focuses on the first topic. The interview guide was reviewed and adjusted after conducting three pilot interviews that were included in the analysis.

Interviews were conducted face-to-face (with the exception of one interview that was conducted via the application Zoom, according to the participants’ request) in Swedish (*n* = 10), English (*n* = 4), and Arabic (*n* = 4). All interviews were audio-recorded and lasted on average 25 min, with interviews conducted with participants who self-identified as women lasting longer and were up to 53 min. Information regarding participants’ sociodemographic characteristics (gender, age, length of stay in Sweden, country of origin, languages spoken at home, and self-reported household's financial situation) was collected via an anonymous pre-interview questionnaire (Appendix B).

### 2.4 Data analysis

The data were analyzed using reflexive thematic analysis (Braun and Clarke, 2021; Braun and Clarke, 2019). First, the interviews were transcribed verbatim, transcripts were read several times to familiarize with the data, and notes were taken. Second, sections related to experiences with SRH services and information were identified and coded inductively by the first author using the software NVivo (released in March 2020). Third, when half of the interviews were coded, the first author started generating preliminary thematic patterns in the data. Throughout the coding process memos were taken. These memos consisted of preliminary interpretations and analytical impressions of the data and were used along with the notes when generating preliminary thematic patterns and initial themes. This step also involved investigator triangulation (Guion et al., 2011): three interviews were coded separately by the authors who also developed a list of preliminary thematic patterns separately. Codes and preliminary thematic patterns were compared in a consensus session, which allowed for confirming the preliminary findings. Fourth, after coding the rest of the interviews, initial themes and sub-themes were developed by the first author. These initial themes were discussed, reviewed, and refined by all authors, and this step led to developing the three final themes presented in this paper (Appendix C).

### 2.5 Ethical considerations

The Swedish Ethical Review Authority approved the study (Dnr: 2020–01,043 and Dnr: 2022–04,725–02). Verbal informed consent for interviews and audio recordings were obtained from all participants. Prior to the interviews, a consent form was provided to the participants. In addition to information about the study aim and procedure, the consent script included contact information for counseling services to be accessed in case of emotional distress or discomfort felt during the interviews. Participants were informed that they had the right to withdraw at any time. After reviewing the ethical application in November 2022 (Dnr: 2022–04,725–02), it was possible to provide movie tickets to the participants after the interviews (one movie ticket each) as a compensation for their time and participation. Only two interviews were conducted after the change, and the participants received their compensation. Identifying information such as participants’ names, schools, and place of residence were removed during the transcription, and pseudonyms are used in this paper to protect participants’ identity. Some pseudonyms were chosen by the participants, while others were assigned by the first author.

## 3 Results

### 3.1 Study population

Most participants^1^ self-identified as men (12 out of 18). The majority of the participants’ age ranged between 17 and 25 at the moment of conducting interviews with six participants were aged <20, and only one participant was aged 26. Participants’ length of stay in Sweden ranged from two months to 16 years, and 13 out of 18 participants were high school students or college students. Half of the participants spoke more than one language at home, and more than half (10 out of 18) described their families’ financial situation as poor or about average.

### 3.2 Themes

The analysis yielded three themes that reflect participants’ accounts of experiences when accessing SRH services and information. The first theme *SRH services: dual perceptions and experiences* captures participants’ perceptions of SRH services, namely youth clinics, and their experiences of accessing SRH services. This theme also addresses participants’ understanding of factors affecting their use - or non-use - of these services. The second theme *Sexuality education: an eye-opener or a joke?* reflects participants’ experiences of receiving sexuality education in schools. The third theme *SRH information: beyond formal services and education* explores participants’ ways of accessing SRH information that goes beyond information provided by various SRH services and formal sexuality education in schools.

#### 3.2.1 SRH services: dual perceptions and experiences

Participants shared ambivalent perceptions of SRH services. They mainly discussed their perceptions of the youth clinic which was considered the most common destination for seeking SRH services among them. Participants shared either past or current negative views towards these clinics. Within these negative views, the youth clinic was perceived as a scary and stigmatized place associated with shame and discomfort, as well as with SRH concerns or risks such as unwanted pregnancy and STIs. When describing her first perception of the youth clinic, Lara said:

“I didn't hear about it (the youth clinic) from school. But I think that my friends were the ones who mentioned it; and for me, it *was the scary place…like my friends will go there if they have problems…”*

Some participants reflected on these negative perceptions and related them to different factors including stigma surrounding sexual activity, being young, as well as general distrust regarding the youth clinic, its activities and role. For example, Alaa recalled his first encounter with the youth clinic in the eighth grade, which was part of a study visit organized by the school. He described how he and his peers were lacking enthusiasm for the visit by saying: *“We didn't take them seriously”.*

As explained by Alaa, this attitude stemmed from a pre-understanding of the youth clinic's role that was disapproved by him and his peers:

*“And then there are also bad rumors about the youth clinic. You know they can interfere between families…and you know, when kids do something that the parents don't know about, kids can run to [the youth clinic]. They have birth control pills and all that. That's their job. But when you are a child, you also hear such things, you don't like it”.*

Participants’ perceptions of the youth clinics were not static, but rather shifting over time. Hala described how her perception of the youth clinic changed over time by saying:

*“So, obviously going there for the first time was terrifying. But after that, it felt like it was a safe space. I feel like after you go there and see how it is, you might feel more comfortable, and you feel safer. But the idea of it is scary because of the stigma around sexual relations in general, especially at young age.”*

In contrast to the negative perceptions, the youth clinic was viewed as a safe space by some participants. They expressed their appreciation for having a specific place as youth or young adults for seeking SRH services and accessing understandable SRH information. They also expressed their satisfaction with the privacy measures at youth clinics. In this sense, the youth clinic seemed to convey a feeling of security and safety to these participants. Saif highlighted an important preventive role of youth clinic by saying:

*“I think it is good that it [the youth clinic] exists. It reduces the risk that when you are young and would get someone pregnant and so. So, you can go there and get condoms; or if you need an abortion or something, so you can go there and talk to them; or if you have problems or something like that, you can always call them and talk to them”.*

Most participants reported not having an experience with using youth clinics or other types of SRH services (e.g., primary healthcare centers) or the webpage of the youth clinics, and they shared different explanations for this. Although many participants were aware of the services provided at the youth clinic, some, namely newly arrived participants, shared their lack of knowledge about these services. Additionally, some participants mentioned how the negative perceptions of youth clinic led them to avoiding seeking help there. Others felt that they did not need SRH services and considered that these services were not relevant for them as they were not sexually active. Seeking SRH services was perceived by some participants as not only uncomfortable, but also as a taboo. According to Lara, this taboo might be more pronounced among youth with migration experience:

*“I think it's probably scarier for immigrants or migrants just because like…even if your family is open minded, even if it's like more, let's say, stereotypically more Swedish than let say, Middle Eastern. It is still…a taboo in one way or another…like… it will always be a taboo…”*

The difficulties in accessing the youth clinic were also brought up by some participants as an explanation for their restricted use of SRH services, and different dimensions of access to youth clinics were described. One such dimension was physical accessibility. Rima felt that the youth clinic in her city was a hard-to-find location. She discussed the difficulty of ensuring a balance between enhancing the physical accessibility of the youth clinic and protecting youth privacy and confidentiality:

*“I don't think all know where these clinics are. I had personally…like first time helping a friend out, we had a very hard time finding where the clinic is. (…) And I think just be more accessible…that would be a very good, a good thing for a lot of students, and a lot of young adults, to know where it is. (…). It's very controversial. It's a very hard question (…) like a lot of people will be like because they don't want to be noticed…like some people don't want to know that others have done something that is taboo, I think; and having it public might make it worse for these people, and they won't search for help maybe……”*

In addition to the physical location, Tara emphasized the importance of ensuring the flexibility of access to services through, for example, providing drop-in appointments and collaborating with other healthcare facilities by saying:

*“I think they could have… maybe they already have, I'm not sure if I know about it. But for example, drop-in times because I feel that certain things, in certain situations, (…) you could do it a little faster and in a shorter time…You could talk to someone maybe for five minutes or something. This can surely be improved. And for some things, that you may not need to go there. For example, if you want a birth control pill, you could have them in some way, that they would be connected to a pharmacy or something so that you don't have to go to the youth clinic.”*

Despite the low use of SRH services, the participants who were in contact with the youth clinics generally described positive encounters with the providers. They expressed their satisfaction with the services and help they received, and highlighted healthcare providers’ responsiveness and openness. Tara recalled her experience of seeking services related to STIs information and testing together with her ex-boyfriend by saying:

*“It was great…very short but clear. He [the medical doctor] helped us understand if there is no symptom, there is nothing to worry about. If I haven't had unprotected sex, for example, then there's really no need [for testing]. But if I feel like doing it, that's absolutely fine. (…) That was so helpful, I would say. I understood well.”*

Positive encounters were not restricted to the youth clinic. A similar experience was reported by Marwan who recently moved to Sweden and accessed SRH services at a healthcare center. He described the interaction with healthcare providers as friendly and shared how he felt safe in the healthcare center as both the privacy measures and the perceived LGBT friendliness contributed to a sense of safety as expressed below:

*“The way they treated me was very nice…and the encounter was private and no one bothered us. It was very friendly to LGBT people, and they even talked to an interpreter to explain more to me what I should do in the center. I felt the way I was treated was good. As soon as you are in the healthcare center, you can see a rainbow flag… that they are LGBT friendly. It made me feel a little safe.”*

Few participants recalled negative experiences when using SRH services. While the majority appreciated the privacy measures at the youth clinic, Samar recounted a different experience. She shared experiencing what she viewed as a breach of confidentiality when healthcare providers reported a past sexual harassment incident to her father. This event resulted in eroding the trust between the healthcare providers and Samar who decided to stop using the youth clinic. She voiced her discontent by saying:

*“When they [the youth] seek help, they want someone they trust…100 percent; that their parents can't find out anything. It should be completely confidential, even for youth who are under 16. I mean they are not adults yet, but if they say I don't want my parents to find out about this thing. So, it shouldn't happen because they have trusted these people.”*

Another participant, Sana, who was an undocumented asylum seeker expressed her dissatisfaction with SRH services provided, and shared how she felt not being taken seriously by healthcare providers as she consulted healthcare centers for menstrual pain several times:

*“They [healthcare providers] said it was normal. I just need to take some pain killers. But I had so much pain. It was so painful that I couldn't sleep…I wanted to get examined to see if maybe I had an inflammation or something wrong with my ovaries… (…) I went several times, and no one cared (…). Maybe now that I got residence permit, I can go to the doctor and get examined…”*

#### 3.2.2 Sexuality education: an eye-opener or a joke?

Sexuality education represented one of the main sources of SRH information for the participants. Most had received school-based sexuality education in Sweden and shared different attitudes towards it. Some felt that sexuality education was an eye-opener. It was described as a way to learn about the body, normalize being sexually active, receive SRH prevention-related information, understand consent, and prevent sexual abuse. They also expressed gratitude for receiving sexuality education in school. Rima said:

*“I am grateful that we got that in class. It is important to understand how sexually transmitted diseases happen…how you can protect yourself from these kinds of things, how for you to have sex there needs to be a consent. A lot of people don't know that because they never really talked about it. It's just sex. I will just do it. But when you get to understand that there has to be a clear yes, and no is always a no You get to learn that from these classes.”*

When discussing the importance of sexuality education, some participants highlighted its role in challenging what they perceived as a bad or distorted image of sex found in pornography. Salem shared:

*“But I actually think it is necessary and important that everyone understands what it [sex] means, and that you get a picture of it, a correct picture of course, of what it is because you can also have a bad picture, from a lot of pornography.”*

Sexuality education was, however, not well received by all participants. Some, mainly participants who self-identified as men, recalled negative attitudes and feelings towards sexuality education classes in school. They described how they felt ashamed and uncomfortable in the classroom and shared how they used joking to cope with such feelings. Some also shared that they did not take sexuality education seriously, and described how they were not in engaged in the classes, as expressed below:

*“We were all in class. But I don't remember what they said. It was… I remember we laughed a lot. When you were little, you would laugh at such things…” (Alaa)*

Participants also pointed to low acceptance of sexuality education content, by either their fellow students or their parents. A few reflected on their previous experiences and discussed explanations for their negative attitudes towards school-based sexuality education. Disapproval of sexuality education content, early age or lack of maturity, and religion were brought up as explanations for these attitudes. Lara said:

*“I know, for instance, a lot of girls in my class…they miss the sex education a bit because they were like “(…) “Oh! This is not OK because these are naked people. I don't want to see them.” So, they missed it.”*

When recalling the experience of receiving sexuality education in school, Amir contrasted attitudes of Swedish students and students with a migrant background. According to him, Swedish students were more receptive of sexuality education compared to students with a migrant background and he made a clear link between normalizing sexuality education, talking about sex, and Swedishness:

*“In ninth grade, I moved to a more Swedish school. It was really Swedish; and it was completely normal for them to talk about such things [sex]. So, when we had classes [sexuality education] like that, they were completely normal about talking about things like this (…). And when I heard that in ninth grade, I was kind of shocked. I've never been able to do that.”*

Some participants felt that the sexuality education provided in school was useful yet lacking in comprehensiveness. They criticized the dominant biological approach that might not capture the multidimensionality of sexuality. Tara described the sexuality education received in school by saying:

*“Quite informative, especially what they have it in school… Apart from that, it was specific. It was a lot of biology. It was connected to the subject of biology (…) But I think I've always felt that there's so much more to talk about; and it's especially with the part that what is sexuality (…). Everyone is different; and sex is so different for everyone (…). I think that's the most important”.*

Other participants viewed the sexuality education that they received as heteronormative and lacking practical information about relations and sexuality. For instance, Hala noticed:

*“I feel, for example, they didn't really talk much about anything but heterosexual sex, for example…”*

A few participants reflected on the pedagogical approach adopted when providing sexuality education, mainly on the approach of separating boys and girls in different classrooms and providing different information deemed more relevant for each gender. Participants felt that this approach had resulted in knowledge gaps about other genders’ sexuality and needs. Hala shared:

*“I mean one thing which I think it wasn't very effective, or not great, is that they separate. I mean they separated us into girls and boys…and obviously, the girls had maybe more focus on periods and stuff like that, but maybe the guys didn't. But I don't think that was a very good idea because I think both genders should be kind of educated on the same level regarding both matters, in my opinion”.*

#### 3.2.3 SRH information: beyond formal services and education

Participants discussed other sources of SRH information, sources that were found beyond the one provided by the formal SRH services, namely the youth clinics, and school-based sexuality education. These sources include the family, friends, and the internet. A few participants mentioned that they had conversations about sex or sexuality or relationships with their parents. Some mentioned that they had such conversations with only their mothers, whom they perceived as particularly open. Rima said:

*“It's very nice to be surrounded by people that are willing to be open about it, and so was my mom. She was very open about teaching us and being part of the society where you understand that at some time of your life, you will be interested in some kinds of things, and that it's ok, but you need to protect yourself; and explained how to protect yourself.”*

Contrarily to the few participants who had some sort of open communication about sex and sexuality with their mothers, most participants mentioned that they had never talked about sex with their parents. Feeling uncomfortable discussing such topics with parents or adults in general as well as the family's cultural and religious background were brought up as explanations for this lack of communication. Ibbe stated:

*“We don't have that kind of conversation, not about sex and condoms. We in the family are religious.”*

Nearly all participants described how they usually had conversations about sexuality with their friends. Friends were perceived as a safe space for these conversations. Nevertheless, the degree of openness and the extent of sharing experiences and information among friends varied across participants. Similarly, the meanings attached to these conversations differed. Some found that these conversations were an opportunity to access information about different aspects of SRH including information about SRH services, and prevention methods such as condom use and consent. Others felt that although generally open, these conversations did not level up to being a proper source of knowledge or even a serious discussion. Walid shared:

*“So, it's not a problem with friends. It's not a problem actually. It's not a problem to talk to friends because, you know, we're still close… So, it is open between us. We can talk about it. (…) When we talk to each other, we joke…we laugh about it. But it's nothing so direct…”*

Participants’ informal sources of knowledge about SRH, sex, and sexuality as well as their perceptions of these sources varied substantially. Some mentioned seeking SRH information on the internet, while others questioned the reliability of such information. Hassan considered the internet as an important source of SRH information and described his strategy of checking this information by saying:

*“I never trust a single (…) I never just search on a single website. I search on more and more websites until I get similarities between all the answers to the question because everyone has his own opinion on something. So, I check all of them; and when I have many similarities, I'm sure that this is the right answer.”*

Additionally, despite the general skepticism towards pornography, a few participants mentioned that they or their peers used pornography to learn about sex. Amir said:

*“They kind of started like that, classmates in the hall, they started watching porn and stuff like that. Do you get it? Just to learn…because of what they had listened to in the class. So, they learned, and I didn't know what it was. Then, when they started looking at it, I also started going at home, in the evening, looking at it like them”.*

Moreover, a few participants who were newly arrived migrants mentioned relying on SRH information provided by non-governmental organizations as it was the first point of access to this information.

## 4 Discussion

The study highlights the ambivalent perceptions and experiences of young people with migration experience in relation to SRH services. It also sheds light on the positive and negatives experiences and attitudes among participants when receiving school-based sexuality education along with discussing other sources of SRH information.

The findings show how the interplay between participants’ negative perceptions of youth clinics and the difficult access to these clinics contributed to low service use. This low use is exacerbated by the perception of irrelevancy of SRH services to many young people interviewed in this study. The study findings are in line with previous research conducted in Sweden and other high-income countries (Salad et al., 2015; Afroz et al., 2021; Botfield et al., 2020; Baroudi et al., 2020; Tirado et al., 2022; Waenerlund et al., 2020). For instance, a survey study assessing the friendliness of youth clinics in Northern Sweden found that youth born outside of Sweden reported less satisfaction with access to these services compared to other youth groups (Waenerlund et al., 2020). By applying the concept of sexual citizenship (Weeks, 1998; Richardson, 2000; Talburt, 2018; Richardson, 2018), the study indicates that young people with migration experience do not access and use SRH services to the degree necessary for achieving SRHR and thereby enjoying full sexual citizenship.

Previous research conducted in Sweden pointed to different explanations for the low use of SRH services among young people with migration experience including lack of knowledge about available services, lack of trust towards the Swedish system, as well as language and financial difficulties (Baroudi et al., 2021; Baroudi et al., 2020; Tirado et al., 2022). This study adds to this research by highlighting additional explanations, i.e., associating youth clinics with shame and SRH concerns or risks, limited accessibility of these services, and the perceived irrelevancy of these services for some young people with migration experience. The perception of irrelevancy of SRH services among participants who are not sexually active might stem from an understanding that sexuality is equivalent to having sex and that these services are primarily targeting sexually active youth and young people. Communicating comprehensive conceptualizations of sexuality and SRH among youth might help overcome this perceptual barrier to SRH service use. While this study has highlighted proximal explanations for the challenges to accessing and using SRH services among young people with migration experience, future research should delve into more distal systemic and structural factors and explore how young people's experiences are shaped by interweaving power structures. For instance, Cabieses et al. (Cabieses et al., 2023) argue for applying an intersectional lens in research on health and migration which allows shifting from an individual, culturally based approach to an approach that accounts for the effects of structural factors like migration policies and racialization processes.

While some participants held negative views towards the youth clinic, others viewed the youth clinic as a safe space. Oscillating between perceiving youth clinics as a safe space and viewing reaching out to youth clinics as a “scary experience” was also shared by youth and young adults in a qualitative study conducted in Northern Sweden (Thomson et al., 2022). In that study, some participants viewed going to the youth clinic as “a potentially scary, awkward and vulnerable experience”, whereas the perceived relaxed and hospitable environment at the youth clinic, as well as the healthcare providers’ respectful and friendly attitudes, contributed to a sense of safety (Thomson et al., 2022). In addition to healthcare providers’ openness and responsiveness, having a specific place as a youth or young adult for seeking SRH services conveyed a sense of safety for some participants in the current study.

Although considered an important source of SRH information, sexuality education was not always well received by the participants who were disengaged in the classroom, which might reflect their discontent with its content. While Swedish sexuality education has adopted a norm-critical approach based on challenging and questioning norms in society (Bredström et al., 2018; Bengtsson and Bolander, 2020), some areas might have been left untouched. According to Bredström et al. (2018) the norm-critical sexuality education in Sweden is stamped by the discourse of Swedishness and “is caught up in a neoassimilatory framework where certain sexual values are promoted as universal and uncontested” (29 p. 538). The content of this education might, therefore, not accommodate the needs and perspectives of youth with different backgrounds. This suggests that Swedish sexuality education might have become a contested space for inclusion/exclusion into sexual citizenship. As argued by Alldred and Fox (Alldred and Fox, 2019), sexuality education contributes to the process of sexual “citizen-ing” youth i.e., the social production of sexual citizenship, by shaping their capacities in relation to sexuality and relationships. These capacities, for example, the capacity to assert sexual rights and make decisions in relation to sexuality and reproduction, do not only affect young people's sexualities but broadly their social engagement and participation as (sexual) citizens (Alldred and Fox, 2019).

Furthermore, the current study points to shortcomings of sexuality education voiced by some participants: the heteronormative content, the reliance on single-gender education, and the strong emphasis on the biological aspects of sexuality while overlooking other aspects. While the critiques of heteronormativity and the dominance of the biological approach were raised in previous literature (Lukkerz, 2023), more research is needed to compare the perceptions of single-gender vs. mixed sexuality education among young people and teachers in Sweden. The dominance of the biological approach can be explained by the fact that Swedish sexuality education is rooted in a biomedical framework where biological aspects of sexuality (e.g., pregnancy and STIs) are prioritized (Lukkerz, 2023). This approach is not restricted to the Swedish context as similar critiques were expressed by young people in international research (Pound et al., 2016). These shortcomings suggest that school-based education might fail to meet the complex needs of young people who might seek SRH information elsewhere (Fraser et al., 2021).

In this study, several informal sources of SRH information were discussed by the participants, including family, friends, and the internet. Interestingly, views on the reliability of information provided by friends and the internet varied across participants suggesting that young people with migration experience are not passive consumers of SRH information. As outlined in previous research, young people can critically engage in assessing the reliability and quality of SRH information using strategies, such as synthesizing different sources and creating hierarchies of expertise (Fraser et al., 2021; Farrugia et al., 2021). Further research is needed to explore the strategies adopted by young people with migration experience for appraising SRH information. This research might inform the provision of tailored and relevant SRH information.

### 4.1 Methodological considerations

The findings of this study were drawn from interviews with young people with migration aged between 17 and 26. The average length of the interviews is 25 min as some interviews with participants who self-identified as men were particularly short, which might not have allowed a deep exploration of their experiences. Moreover, the sample composition might limit the transferability of the findings. Despite striving to recruit participants from different areas of Scania County, nearly all participants in this study were living in urban settings. Their experiences might not capture the experiences of young people with migration experience living in rural areas. Additionally, the sample included only participants with a Middle Eastern background. While people with a Middle Eastern background represent one of the biggest migrant communities in Sweden (SCB 2024) and the region where the study was conducted (RegionSkåne 2023), it is worth noting that the accounts of experiences of other groups of young people with migration experience might differ from the ones reported in this article. Therefore, there is a need for future explorations focusing on the perspectives and experiences when accessing and using SRH services of diverse groups of young people with migration experience in terms of, for example, ethnic background, gender, and geographic location. Such explorations might allow for generating a more comprehensive understanding of the SRH-related experiences of young people with migration experience.

Several measures were taken to enhance the trustworthiness of this study (Lincoln and Guba, 1986). Quotations illustrating the final themes were used to enhance the study's dependability and confirmability. To enhance study credibility, investigator triangulation during the coding process (Guion et al., 2011) along with continuous discussions throughout the data analysis process were used.

## 5 Conclusions

The study findings suggest that multiple factors might be involved in the low of use of SRH services, namely the youth clinics, among people with migration experience in Southern Sweden. These factors include associating youth clinics with shame and SRH concerns (e.g., unintended pregnancy, STIs), as well as the difficult access and perceived irrelevancy of these services. Multicomponent strategies to improve the accessibility of these services and counteract negative perceptions are needed. These strategies can include providing outreach youth clinics in areas with a high density of migrants and incorporating the specific needs of young people with migration experience in the provision of SRH services.

The study also highlights different sources of accessing SRH information among young people with migration experience ranging from school-based sexuality education to informal sources (e.g., friends and the internet). Participants’ perceptions of attitudes toward school-based sexuality education were ambivalent. While some participants considered sexuality education as an eye-opener, others described their discomfort and disengagement in the classroom. Extending the norm-critical approach in Swedish sexuality education to challenge norms related to Swedishness and the universality of sexual values might foster the engagement of youth with diverse backgrounds and ensure their sexual citizenship.

## Funding

This research did not receive any specific grant from funding agencies in the public, commercial, or not-for-profit sectors.

## Ethics approval and informed consent

The Swedish Ethical Review Authority approved the study (Dnr: 2020–01,043 and Dnr: 2022–04,725–02). Prior to interviews, a verbal informed consent for interviews and audio-recordings was obtained from all participants. After reviewing the ethical application in November 2022 (Dnr: 2022–04,725–02), it was possible to provide movie tickets to the participants after the interviews (one movie ticket each) as a compensation for their time and participation. Only two interviews were conducted after the change, and the participants received their compensation. All methods were carried out in accordance with relevant guidelines and regulations. The use of verbal informed consent was approved by the Swedish Ethical Review Authority (Dnr: 2020–01,043 and Dnr: 2022–04,725–02).

## CRediT authorship contribution statement

**Nada Amroussia:** Conceptualization, Formal analysis, Investigation, Methodology, Writing – original draft, Writing – review & editing. **Malin Lindroth:** Conceptualization, Methodology, Supervision, Validation, Writing – review & editing. **Catrine Andersson:** Conceptualization, Methodology, Supervision, Validation, Writing – review & editing.

## Declaration of competing interest

The authors declare that they have no known competing financial interests or personal relationships that could have appeared to influence the work reported in this paper

## Appendix Supplementary materials

Image, application 1

## Footnotes

1 Two participants did not fill the pre-interview questionnaire and had missing information.
