# Supplementary material for: Young people with migration experience and their (non) encounters with Swedish sexual and reproductive health services and information: An explorative study
Source: J Migr Health. 2024 Oct 5;10:100270. doi: 10.1016/j.jmh.2024.100270 (PMC11490863; doi:10.1016/j.jmh.2024.100270)
Supplement: Supplementary file 1 [file mmc1.docx]

**Appendices**

**Appendix A-Interview guide**

Thank you for volunteering to take part in this study. I appreciate your time and effort to be with me. My name is .................................... I have some questions about your sexual experiences and your experiences with health services in Sweden, especially sexual and reproductive health services. I mean by sexual and reproductive health services for example SRH *information and counselling, and sexuality education; prevention and treatment of IST including HIV; treatment of reproductive system cancers and breast cancer, abortion and contraception counselling and services, antenatal, childbirth and postnatal care…*

I promise to keep your identity and answers confidential. Before starting the interview, I would like to remind you that there are no right or wrong answers to any of the questions that we ask. All your experiences and opinions are valid. Your participation is completely voluntary. You can stop your participation at any time, and you have the right to not answer or skip questions if you want to. We will audio-record our discussions and the recording will be transcribed. However, only the research team will have access to the recording. We encourage you to use pseudonyms to protect your confidentiality. We will not associate any identifying information (name, place of work…) with the recording or transcripts.

1. Could you present yourself?
2. What made you decide to participate in this study?

**The participants’ experiences with SRH services and information**

1. Have you ever had an experience seeking sexual and reproductive health services? What types of health services have you sought? Could you tell me more about that?
2. Have you ever had an experience using sexual and reproductive health services? What types of health services have you accessed? Could you tell me more about your experience?
3. How do you describe these services?
   1. How do you view these services, for example at the youth clinic?
4. How do you describe your interactions with healthcare providers?
5. Think about the times that you sought help from sexual and reproductive health services, did you get the help that you needed? Could you tell me/us more about that?
   1. If yes, how?
   2. If no, how and why? (Probe is there any difficulty?)
6. In your opinion, what types of sexual and reproductive health services and commodities should be available for you?
7. What type of suggestions do you have to improve your experiences with sexual and reproductive health services?

**Questions 3, 4, and 6 might not be relevant for participants who have never sought and/or used SRH services. For these participants other group of questions will be asked:**

1. If you didn’t seek or use these SRH services, where do you usually seek help in matters related to SRH?
2. What do you think about using these services? (To avoid a direct question “why do you prefer not using these services?”)
3. Where do you seek information in relation to SRH matters?
   1. If participants name some sources: what do you think about these sources?
4. Did you have any experience in discussing SRH matters with your parents or any other family member?
   1. Could you tell me more about this experience?
   2. If not, what make avoid such discussions with your family?
5. What do you think about the sexuality education in school?

**The participants’ views on sexuality and sexual experiences**

1. Some people in your age are exploring new relationships (romantic, sexual…). What do you think about that? Could you tell us/me more about that?
2. In your opinion, what are the conditions or factors that can influence the decision to have sex (sexual relations) among people in your age?
3. What do you think is important when having sexual relations?
4. Do you want to add something?

Thank you for your participation in this study.

**Appendix B-Pre-interview questionnaire**

**Introduction**

We have few questions about your background. Could you please fill this questionnaire? This questionnaire will help us to understand better our discussion. We will not mention your name or any other identifier in our report.

**Pre-interview questionnaire**

| **N°** | **Question** | **Answer** |  |  |
| --- | --- | --- | --- | --- |
| 1 | How old are you?  [Enter age in years] | Years | l__l__l |  |
| 2 | What is your gender? By gender we mean gender identity, i.e., the gender you feel like.  [Circle your answer(s)] | A. Woman  B. Man  C. Non-binary  D. Other alternatives, write here  ………………………………….  E. Unsure  F. Prefer not to answer |  |  |
| 3 | How do you describe yourself?  [Choose all that apply] | A. Swedish  B. Non-Swedish  C. Other alternatives, write here:  ………………………………… |  |  |
| 4 | Were you born in Sweden?  [Circle your answer] | A. Yes  B. No |  | You can continue with question 7 if you answered “yes” |
| 5 | If no, where were you born?  [Enter the name of the country] | ………………………… |  |  |
| 6 | For how long have you been living in Sweden? | Years | l__l__l |  |
| 7 | How many languages do you speak at home?  [Circle your answer] | A. One, only Swedish  B. One, not Swedish  [put the language name here………………….]  C. Two, Swedish and another language  [put the language name here………………….]  D. Two, both are not Swedish  [put the languages’ names here………………]  E. Other  [write here: ………………………………………………………………………………………………………………………………………………………………………] |  |  |
| 8 | What is your highest completed level of education? | A. Elementary school, primary school, or similar  B. 2 years of upper secondary school or high school  C. 3–4 years of upper secondary school or high school  D. Some higher education  E. University or college, less than 3 years  F. University or college, 3 years or more |  |  |
| 9 | What is your present form of employment? | A. Studying, training  B. Work as an employee. Enter percentage of full-time.  C. Self-employed  D. Labor market measures  E. Unemployed  F. Other, write here: ……………………………............................... |  |  |
| 10 | Think about your family when you were growing up, from birth to age 16. Would you say your family during that time was pretty well-off financially, about average, or poor?  [Circle your answer] | A. Pretty well-off financially  B. About average  C. Poor  D. It varied |  |  |

**Appendix C-Analysis process**

| Final theme | Preliminary theme | Preliminary sub-theme | Examples of codes |
| --- | --- | --- | --- |
| SRH services: dual perceptions and experiences | The youth clinic: “It is good that it exists” | Between avoidance and appreciation | - Associating youth clinic SRH concerns  - A stigmatized place  - A safer space  - Importance for prevention  - Changing perceptions |
|  |  | Not much used services | - Not relevant  - Not needed  - Difficult access  - Trust  - Feeling helped  - Appreciating privacy measures |
| Sexuality education: an eye-opener or a joke? | A comprehensive sexuality education? | Ambivalent receptions of sexuality education | - Informative and crucial  - Appreciating sexuality education  - Discomfort at Sex Ed classes  - Disengagement  - Joking as a coping mechanism  - Low acceptance |
|  |  | Still incomplete! | - Dominance of biological approach  - Gender separation  - Gaps in the knowledge  - Omitting sexuality  - Heteronormative |
| SRH information: beyond formal services and education | Beyond formal sexuality education | “The talk”: rarely experienced | - Openness of the mother  - Countering porn influence by sex talk  - Feeling uncomfortable  - Parents as not a source of knowledge  - Relating not having sex talk to religion |
|  |  | Friends: a perceived safer space | - Appreciating sharing information with friends  - Differences in discussing SRH with friends  - Discussing consent with friends  - Feeling comfortable  - A friend as a mediator in receiving information from youth clinics |
|  |  | The internet: not always reliable | -Seeking help on the internet  - Internet information as a source of panic  - Inaccuracy of information on the internet  - Cross-checking  - Watching porn as part of the learning process |
